# Characterization of Epicardial-Derived Cardiac Interstitial Cells: Differentiation and Mobilization of Heart Fibroblast Progenitors

**DOI:** 10.1371/journal.pone.0053694

**Published:** 2013-01-18

**Authors:** Adrián Ruiz-Villalba, Algirdas Ziogas, Martin Ehrbar, José M. Pérez-Pomares

**Affiliations:** 1 Department of Animal Biology, Faculty of Science, University of Málaga, Málaga, Spain; 2 Department of Obstetrics, University Hospital Zürich, Zürich, Switzerland; The University of Hong Kong, Hong Kong

## Abstract

The non-muscular cells that populate the space found between cardiomyocyte fibers are known as ‘cardiac interstitial cells’ (CICs). CICs are heterogeneous in nature and include different cardiac progenitor/stem cells, cardiac fibroblasts and other cell types. Upon heart damage CICs soon respond by initiating a reparative response that transforms with time into extensive fibrosis and heart failure. Despite the biomedical relevance of CICs, controversy remains on the ontogenetic relationship existing between the different cell kinds homing at the cardiac interstitium, as well as on the molecular signals that regulate their differentiation, maturation, mutual interaction and role in adult cardiac homeostasis and disease. Our work focuses on the analysis of epicardial-derived cells, the first cell type that colonizes the cardiac interstitium. We present here a characterization and an experimental analysis of the differentiation potential and mobilization properties of a new cell line derived from mouse embryonic epicardium (EPIC). Our results indicate that these cells express some markers associated with cardiovascular stemness and retain part of the multipotent properties of embryonic epicardial derivatives, spontaneously differentiating into smooth muscle, and fibroblast/myofibroblast-like cells. Epicardium-derived cells are also shown to initiate a characteristic response to different growth factors, to display a characteristic proteolytic expression profile and to degrade biological matrices in 3D *in vitro* assays. Taken together, these data indicate that EPICs are relevant to the analysis of epicardial-derived CICs, and are a god model for the research on cardiac fibroblasts and the role these cells play in ventricular remodeling in both ischemic or non/ischemic myocardial disease.

## Introduction

Cardiac muscle cells (cardiomyocytes) are frequently thought to be the most abundant cell type in the adult heart. However, multiple studies have shown that cardiac chamber walls comprise high numbers of non-myocyte cells. These cells and their milieu (the extracellular space between cardiomyocyte fibers) constitute the cardiac interstitium [1]–[2]. Due to the small relative size of cardiac interstitial cells (CICs) and the enormous contribution of cardiomyocytes to cardiac mass, the proportion of CICs versus cardiac muscle cells in the heart is frequently underestimated. In this regard, recent reports suggest that CICs could represent up to a 65% of non-cardiomyocyte cells in the organ [1]–[3].

The biomedical importance of CICs is illustrated by their massive involvement in the remodeling of cardiac ventricular walls after myocardial infarction, a phenomenon that is characterized by a progressive fibrosis [4]. This ventricular remodeling involves the initiation of an inflammatory response and the mobilization of CICs. Both phenomena have been described as a normal response of the adult heart to damage [5]. Other acquired cardiac diseases like dilated cardiomyopathy are also characterized by fibrotic disorders [6].

As already indicated, adult CICs are a heterogeneous population of cells [7]. The phenotypes of CICs range from the characteristic spindle-shaped profile of cardiac fibroblasts (CF) to the more spherical aspect of resident cardiac progenitor/stem cells [8]. From a molecular standpoint, CICs have been phenotyped and classified into different categories by the expression of fibroblastic markers like DDR-2, FSP-1, HSP47, collagen-I [9], [10]; stemness markers such as c-Kit, CD34, or Sca-1 [11]–[15]; or molecules classically related to cardiac embryonic progenitors like Islet 1 (Isl1) or Gata4 [16], [17].

The origin of CICs is also known to be diverse, as reported sources for these cells include bone marrow-derived circulating cells, perivascular cells, the endothelium/endocardium, and the epicardium [3], [7]. Interestingly, only the epicardium shows a very early and persistent contribution to the cardiac interstitium, starting around midgestation [7], [17]–[19].

The embryonic epicardium is an important tissue in cardiac development. It originates from the proepicardium, a cluster of coelomic cells at the caudal end of the developing heart (E9.0–9.5 in the mouse). Proepicardial cells are transferred to the myocardial surface, where they attach and spread forming a continuous monolayered epithelium, the epicardium [20]. While the epicardial epithelium forms, an epithelial-to-mesenchymal transition (EMT) is initiated, so that part of epicardial epithelial cells transform into a population of mesenchymal, highly invasive, epicardium-derived cells (EPDCs). Therefore, the proepicardial-epicardial-EPDC transition should be considered as an anatomical and developmental continuum.

EPDCs differentiate into coronary endothelial and smooth muscle cells [19], [21]–[23], and interstitial fibroblasts both *in vivo* and *in vitro* [18], [24], [25]. Cardiomyocyte differentiation from epicardial progenitors has only been fully confirmed *in vitro* [24], [26], being the *in vivo* differentiation scenario still under debate [17], [27], [28]. Taken together, these results suggest that cells in the epicardial lineage could indeed have multipotent properties [24], [29], [30].

Despite the biomedical importance of CICs, not many reports have characterized these cells as related to their embryonic origin. This approach is most relevant, as our knowledge on the biology of the embryonic sources of CICs can provide clues to understand the responses of the adult interstitium to stress or pathological conditions (e.g. myocardial ischemia). Since the number of EPDCs that can be retrieved from the embryo is really limited, the use of a tool such as a stable cell line is necessary for detailed molecular and experimental analyses. The main goal of this work is to analyze CICs of epicardial origin using a continuous cell line of epicardium-derived interstitial cells (EPIC) as a model, also comparing its properties to those of native epicardial embryonic derivatives.

Our work provides data suggesting that the multipotent properties of cells in the embryonic epicardial lineage are progressively lost throughout development, and accordingly the EPIC line represents a post-EMT EPDC that can differentiate into myofibroblast-like (smooth muscle-like) and fibroblastic cells, but not into myocardial or endothelial cell types. Our results also indicate that EPICs display a characteristic mobilization and proteolytic program, a finding that is relevant to our knowledge of the structure of adult cardiac interstitium, the definition of a cardiac stem cell niche and the interstitial response to stress or damage. This work opens new avenues for the study of cardiac fibroblast/myofibroblast biology and the analysis of mechanisms leading to cardiac remodeling of the diseased heart.

## Materials and Methods

### Ethics statement

The research on mouse embryonic tissue carried out in this study has been approved by the Ethics Committee of the University of Málaga (Spain) under a specific procedure for the controlled breeding of mice and embryo collection. All the work performed in this study was developed in compliance with the Spanish (LAW 32/2007; RD142/2002; RD1201/2005) and European regulations (Directive 86/609/EEC; Directive 2010/63/EU; Commission Recommendation 2007/526/EC) on the use of animals for scientific research.

### Culture of embryonic proepicardial and epicardial cells

E9.5 mouse (C57BL/6) proepicardia and E11.5 embryonic hearts were dissected in EBSS (GIBCO) using forceps, iridectomy scissors and sharpened tungsten needles. Proepicardia were explanted and cultured on poly-L lysinated coverslips, whereas whole hearts were let to attach to 0.1% poly-L lysine or 0.1% gelatin coated-coverslips. Tissues were let to attach overnight. For whole heart explant culture, hearts were removed after an attachment period of 24 hours, leaving characteristic halo of epicardial cells attached to the substrate. These epicardial cells were cultured for an extra period of 48 hours.

### Generation of the EPIC cell line

The EPIC is a continuous cell line derived from E11.5 mouse embryonic epicardium generated at the University of Málaga (Spain). These cells were primarily extracted from whole E11.5 embryonic hearts as described above. Epicardial cell monolayers were cultured for 24 extra hours in DMEM, Penicillin/Streptomycin (GIBCO) and 1 µg/ml 20-methylcholanthrene (MCA, SIGMA). Purity of primary cultures was assessed by cytokeratin immunostaining (see below). After extensive washing with DMEM, cells were let to grow for 4 weeks in their wells, with new DMEM, 1% fetal bovine serum (FBS, PAA) and Penicillin/Streptomycin added every two days. After confluence, cells were trypsinized, replated and cultured at high concentration in DMEM, 10% FBS and Penicillin/Streptomycin. The EPIC line has been growing in culture for more than 3 years.

### EPIC culture dynamics

For regular culture, EPIC were maintained in high glucose DMEM supplemented with 10% FBS, 100 U/mL of penicillin, 100 µg/mL streptomycin and 25 µg/mL of plasmocin (INVIVOGEN), and routinely passaged at confluence.

To plot the growth curve, 10^4^ cells were plated in 100 mm diameter Petri dishes for 10 days; each day 3 dishes were trypsinized and the number of cells was estimated from the suspension in a Neubauer chamber.

### Differentiation assays

To promote the differentiation of embryonic epicardial progenitors (E9.5 proepicardial cells), E11.5 epicardial cells and EPIC (1.6×10^4^ cells/well), samples were cultured in high glucose DMEM supplemented with 1% FBS, 100 U/mL of penicillin and 100 µg/mL streptomycin for 24 hours. All samples were cultured for 24 extra hours in two different media conditioned to promote cell type-specific differentiation: 5% FBS for smooth and cardiac striated muscle; 50 ng/mL bFGF (R&D)+100 ng/mL VEGF-A (R&D) for vascular endothelium.

### Immunohistochemical characterization

Cells were fixed in 70% methanol, Dent's fixative (methanol∶DMSO, 4∶1) or 4% paraformaldehyde, hydrated through a 70%, 50%, 30% ethanol series, extensively washed in PBS, permeabilized and blocked in 5% normal goat serum, 1% bovine serum albumin (BSA) and 0.5% Triton X-100 in Tris-PBS (SBT). Then, cells were incubated overnight in the primary antibody diluted in SBT [1∶100 α-SMA (SIGMA); 1∶20 MF20 (DSHB); 1∶50 SM22 (SIGMA); 1∶100 smooth muscle myosin (Biomedical Technologies); 1∶100 Pan-cytokeratin (DAKO); 1∶100 Pan-Cadherin (SIGMA); 1∶50 CD31/PECAM (BD Biosciences); 1∶50 VEGFR-2 (BD Biosciences); 1∶50 ZO-1 (DSHB), 1∶50 Collagen I (Calbiochem); and 1∶100 FSP-1 (a kind gift from Dr. Eric G. Neilson)]. Samples were washed in PBS and incubated again in a secondary TRITC, Cy5 or FITC-conjugated anti-mouse IgG (SIGMA) at 4°C (8 hours). After final washes in PBS, cell nuclei were counterstained with DAPI (SIGMA) and samples were analyzed under a SP5 laser confocal microscope (LEICA).

### Flow cytometry

Subconfluent EPICs were trypsinized, counted and harvested after a brief spinning. Subsequently, cells were washed once in ice cold binding buffer (PBS with 1% BSA and 0.02% sodium azide) and re-suspended in the same buffer at 2×10^6^cells/ml. The cells were then aliquoted (100 µl), incubated with 0.5 µg primary antibodies at 4°C for 30 minutes, washed twice with binding buffer, and finally incubated on ice and in the dark with 0.25 µg FITC-conjugated secondary antibodies for 30 minutes. Cells were washed twice with binding buffer and analyzed on a FACScalibur cytometer (Becton Dickinson). Incubation with isotype IgG control antibody was used as a negative control. All the antibodies used for FACS were purchased from eBiosciences, except those against VEGFR1&2, EphA1-8, EphB2-4, ephrin A1,2&4 and ephrin B1&2 (all from R&D).

### Semi-quantitative PCR characterization

Total RNA from proepicardial, E11.5 embryonic epicardial cells and EPICs was extracted using the NucleoSpin RNA XS kit (Macherey-Nagel) and submitted to reverse-transcription using oligo dT18 (First Strand cDNA Synthesis Kit/AMV, ROCHE). Ribosomal18S RNA was amplified as reference gene. Analysis of gene expression was carried out using the QuantiTect Primer Assays (Qiagen). The references for the specific primers are: 18s (Mm_Rn18s_2_SG); α-SMA (Mm_Acta2_1_SG); Collagen-I (Mm_Col1a1_1_SG); Mef2c (Mm_Mef2c_1_SG); prolyl-4 hydroxylase (Mm_P4hb_1_SG); SRF (Mm_Srf_1_SG); Scl/Tal1 (Mm_Tal1_1_SG); Tnnt2 (Mm_Tnnt2_1_SG); γ-SMA (Mm_Actg2_1_SG); Gata4 (Mm_Gata4_1_SG); VEGF-R2 (Mm_Kdr_1_SG); Nkx2.5 (Mm_Nkx2-5_1_SG); CD31/PECAM (Mm_Pecam1_1_SG); Wt1 (FW.5′-ATCCTCTGTGGTGCCCAGTA-3′; RV.5′-CGACAGCTGAAGGGCTTTTC-3′); Tcf21(FW.5′-GGCCAACGACAAGTACGAGA -3′; RV. 5′-GTTTGCCGGCCACCATAAAG-3′); Sox9 (FW.5′-AGGAGCACTGAGTCCTTTGC-3′; RV. 5′-CTATCCACGGCACACACACT-3′).

### Quantitative PCR analysis

RNA was extracted from EPIC using TRIzol reagent (SIGMA) as described by manufacturer. RNA concentration was measured with NanoDrop, and 1 µg was submitted to reverse transcription using oligo dT18 (First Strand cDNA Synthesis Kit/AMV, ROCHE). 10 ng of cDNA was used for real-time PCR with specific primers, Maxima™ SYBR Green/ROX qPCR Master Mix (2×) (Fermentas) and BioRad CFX96 Cycler. References for primers are: MMP11 (Mm_Mmp11_1_SG), MMP14 (Mm_Mmp14_1_SG), ADAM10 (Mm_Adam10_1_SG), ADAM15 (Mm_Adam15_1_SG), ADAM17 (Mm_Adam17_1_SG), ADAM19 (Mm_Adam19_1_SG), TIMP1 (Mm_TIMP1_1_SG), TIMP2 (Mm_TIMP2_1_SG), TIMP3 (Mm_TIMP3_1_SG). GAPDH was used as reference gene (Mm_Gapdh_3_SG).

### Matrix degradation and sprouting/proteolytic assays

EPICs were cultured as previously described. Cloning of the EPIC line was carried out by limiting dilution of the stock on 96-well plates (CORNING). 8 different single clones were selected by their characteristic phenotype and growth rate (cEP1–8). Cells were re-suspended in DMEM (GIBCO) supplemented with 10% FBS, 100 U/mL of penicillin and 100 µg/mL streptomycin and mixed with 20% methyl cellulose (SIGMA). Then, 30 µl drops containing an average of 750 cells per drop were distributed over the surface of Petri dishes that were incubated (5% CO_2_, overnight) for a classic hanging drop culture. Between 20–30 spheroids were used per treatment in each experiment. The formed cell spheroids were inspected, photographed with a Leica microscope and removed from plates by gentle washing with 5 ml 1% BSA in PBS. Cell spheroids were centrifuged for 5 min at 600 rpm and resuspended in TBS (20 mM Tris pH7.5; 150 mM NaCl). To proceed with the sprouting/proteolytic assay, 500 µl of regular 3D fibrin gels (5 mg/ml) were formed on the bottom of 24well cell culture plates by the addition of 0.2 U/ml thrombin [31]. Then, a second fibrin gel layer including transglutaminase bound (TG-bound) growth factors [TG-BMP2 (1 µg/ml) and TG-VEGF121 (100 ng/ml)] or soluble factors [bFGF, Wnt3a and Wnt5a (100 ng/ml)] was used to seed EPIC spheroids on top of the first gel layer. TG-binding of growth factors to the fibrin gel allows for the covalent attachment of these molecules to the matrix. Spheroids were photographed after 3 h, 12, 24, 48 and 72 hours time intervals. The digested and/or sprouting area was calculated using Olympus software and plotted as square micrometers. Quantification of differences in sprouting and proteolysis was performed for each individual clone by compiling the spheroid occupied area/digested area when projected into a 2-D image.

For zymography assays, cEP4, EPIC and HT1080 cells were cultured in 100 µl fibrinogen gels (0.3×10^6^cells/gel) supplemented with 200 µl DMEM (without serum). After 24 hours the medium from each culture was collected, centrifuged to remove cell debris and mixed with 4× loading buffer (0.4 M TrisHCl, pH 6.8; 20% glycerol; 0.03% bromophenolblue). 20 µL samples were loaded in each lane and MMP activity was analyzed using gelatine zymography (48 hours). Briefly, 1.5 mg/ml final concentration of gelatin (AppliChem) was added to a 10% standard Laemmli acrylamide polymerization mixture. Human plasmin (FLUCKA) or supernatant from HT1080 cells were loaded as controls. After electrophoresis, gels were incubated twice in 2.5% Triton X-100 (30 min) and then in the zymograpy buffer (50 mMTrisHCl pH 7.5; 200 mM NaCl; 5 mM CaCl_2_; 0.02% NaN_3_; 37°C). Gels were stained with Colloidal blue staining solution (Invitrogen) following the instructions of the provider. Proteolytic activity was visualized as white bands against a blue background. An alternative, independent experiment to test the proteolytic activity of cEP4 was performed culturing these cells in 3D fibrin gels (DMEM/12+1% ITS, no serum) and treating experimental groups with the protease inhibitor aprotinin (1 µg/ml).

## Results

### Generation and characterization of the EPIC line

**Figure 1:**
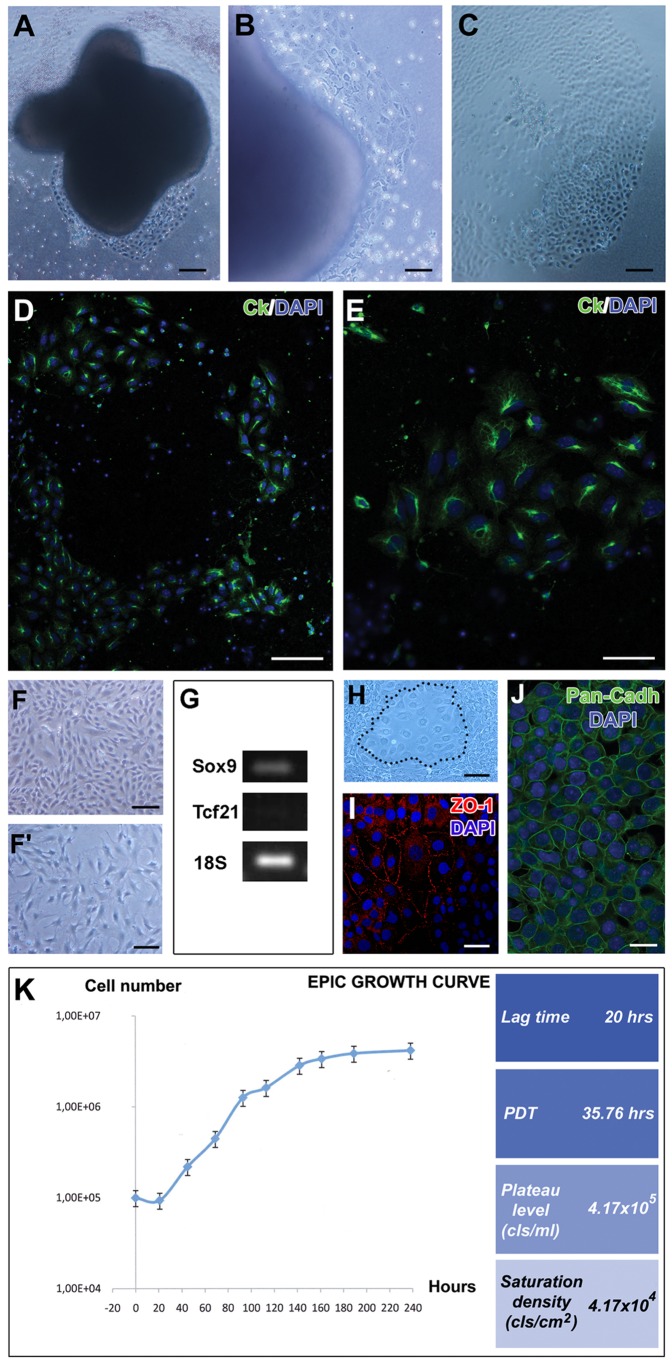
EPIC generation and characterization. **A–C**. Primary culture of E11.5 embryonic epicardium. **A**. Whole heart culture. **B**. Detail showing the outgrowth of epicardial cells from the explanted hearts. **C**. Epicardial cell halo growing on gelatin-coated coverslips. **D,E**. Epicardial cells normally express cytokeratin, a marker for epicardial cells. **F**-**F′**. The majority of EPICs display a mesenchymal phenotype (**F**, confluent culture; **F′**, subconfluent culture) and express Sox9, a known marker for epicardial mesenchymal cells. However, EPICs do not express Tcf21 (**G**). A few, small epithelial-like cell clones (**H**, dotted line) are found dispersed in the culture. Cells in these clones express the epithelial markers ZO-1 (**I**) and cadherins (**J**). **K**. EPIC growth dynamics. The graph shows the parameters defining EPIC cell growth in culture (lag time; population doubling time; plateau level; and saturation density). Scale bars: A,C,D = 100 µm; B,E,F,G = 50 µm; H = ; I,J = 20 µm.

After 48 hours of culture, E11.5 epicardial embryonic cells grew over the substrate and formed a characteristic halo around the explant (Fig. 1A–C). This halo of epicardial cells expressed high levels of cytokeratin (Fig. 1D–E). After incubation with methylcholanthrene (MCA) and sustained culture for one month intense proliferation of primary epicardial cells was recorded. Between the first and third passages the majority of these cells had acquired a mesenchymal phenotype, Continuous passage of cells led to the stabilization of the EPIC line, which have been continuously growing in culture for more than three years.

EPICs were found to have a morphologically heterogeneous appearance; the majority of the cells displayed a mesenchymal phenotype (Fig. 1F,F′) a result that was partially supported by sqPCR analysis of epicardial mesenchymal markers (Sox9 and Tcf21, Fig. 1G). However, a minor number of EPICs grew closely associated when cultured at low densities thus resembling epithelial cells (Fig. 1H). The epithelial-like nature of these small clones was confirmed by immunohistochemistry using ZO-1 and anti-Pan-cadherin antibodies (Fig. 1I,J). In parallel, we evaluated the growth capacity of EPICs and plotted it into a growth curve (Fig. 1K). Our study indicates that EPICs have a short lag state (20 h), suggesting a good adaptation to *in vitro* culture growth, a log phase with a reduced initial growth rate followed by a faster one (see Fig. 1K), and a stationary phase characterized by a slow but continuous cell division, indicating that EPIC do not present contact-dependent inhibition of growth.

### EPIC differentiation potential

**Figure 2:**
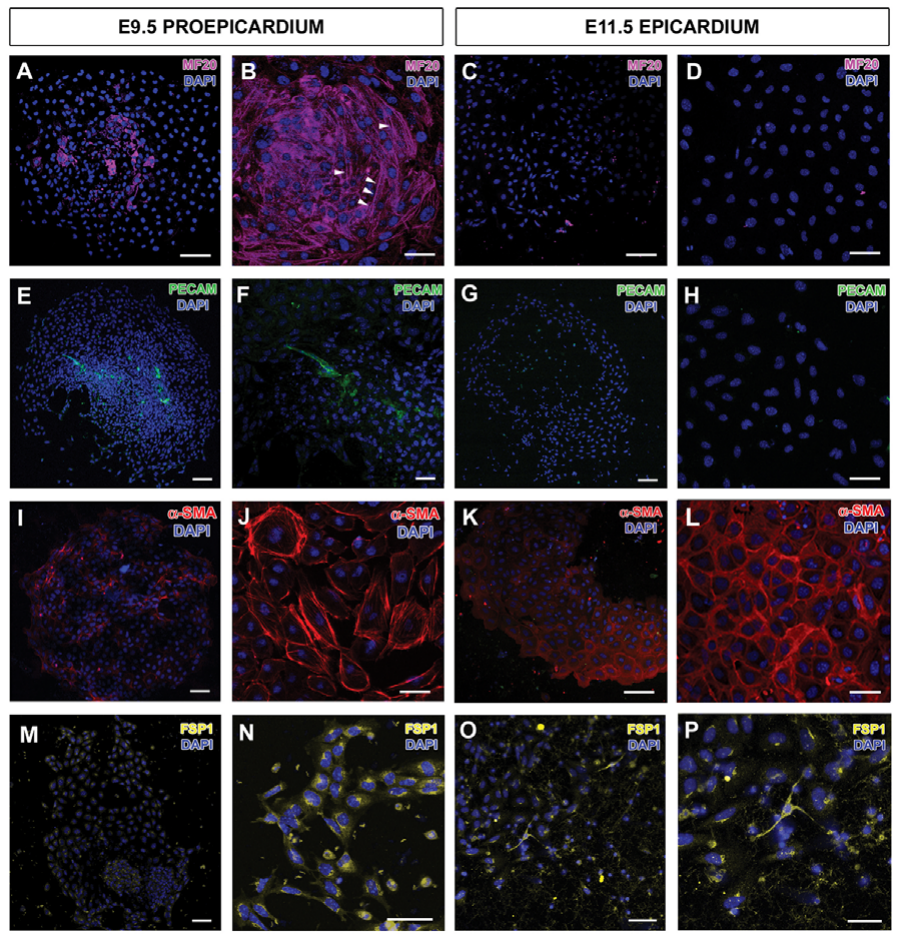
Differentiation potential along the proepicardium-epicardium transition. Proepicardia cultured *in vitro* express differentiation markers for striated heart muscle (MF20, **A**, **B**), endothelial progenitors/cells (**E**, **F**), smooth muscle cells (**I**, **J**) and fibroblasts (**M**, **N**). E11.5 epicardial cells do not express myocardial (**C**, **D**) or endothelial markers (**G**, **H**), but continue to express smooth muscle (α-SMA, **K**, **L**) and fibroblastic ones (FSP-1, **O**, **P**). Scale bars: A,C,E,G,I,K,M = 100 µm; B,D,F,H,J,L,N,O = 50 µm; P = 25 µm.

As already indicated, epicardial embryonic progenitors (the transient proepicardial cells) are known to display multipotent properties both *in vivo* and *in vitro*, although it is not well known whether this multipotency is totally or partially retained by their derivatives [24], [25]. We have therefore compared EPIC differentiation potential to that of embryonic proepicardial (E9.5) and epicardial (E11.5) primary explants, focusing on the four principal cell fates described for embryonic EPDCs (endothelium, smooth muscle, cardiomyocytes and fibroblasts) [18], [19], [24], [31]. Using different growth factor-supplemented media (see Material & Methods) followed by immunohistochemical characterization, cultured proepicardial cells were shown to differentiate into cells expressing myocardial (sarcomeric myosin), endothelial (CD31), smooth muscle/myofibroblast-like (α-SMA) and fibroblastic (FSP-1) cell markers (Fig. 2A,B,E,F,I,J,M,N). E11.5 epicardial explants did not express proteins of differentiated cardiomyocytes (Fig. 2C,D), nor endothelial antigens (Fig. 2G,H) in our explant system; the majority of CD31+ subepicardial coronary vascular progenitors cells and endothelial cords did not migrate from the whole heart explants onto the cell culture substrate (Fig. S1A,B). However, the expression of α-SMA (Fig. 2K,L) and the fibroblastic antigen FSP-1 (Fig. 2O,P) was conspicuous in these E11.5 embryonic epicardial cells.

**Figure 3:**
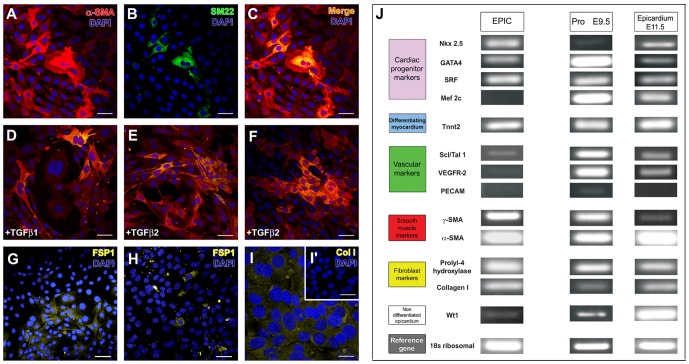
EPIC differentiation marker expression. **A**–**C.** EPIC express α-SMA (red) and SM22 (green). **D–F** Treatment with TGFβ1,2 does not altere the number of cells expressing these two markers, but affects the phenotype of the cells which spread and elongate in culture. EPICs also express fibroblast protein markers like FSP-1 (**G**,**H**) and Collagen I (**I**; **I′** shows the negative, non-inmune control for collagen I immunohistochemistry). **J**. sqPCR profiling. EPIC (left column), E9.5 proepicardium (middle columns) and E11.5 epicardium (right column). Scale bars: A,B,C,D,E,F,H,I′ = 65 µm; G = 100 µm; I = 10 µm.

EPICs consistently expressed specific markers for myofibroblasts (α-SMA, 68.2%), smooth muscle cell (SM22, 21.9%) (Fig. 3A,F, Fig. S1E, Fig. S2), and fibroblasts (FSP-1, 18.5%; Collagen I, 42.2%) (Fig. 3G–I), but are negative for endothelial cell (Fig. S1D; compare to the control VEGFR-2 staining in Fig. S1C) or cardiomyocyte markers (not shown). Treatment of EPICs with TGFβ1,2 did not significantly alter the number of α-SMA+ or SM22+ cells (Fig. 3D–F), but had an impact on gene expression levels (α-SMA and γ-SMA for TGFβ1 and only γ-SMA for TGFβ2, Fig. S2) and morphology of the cells, which spread over the culture displaying long, apparent filopodia and lamellipodia (Fig. 3D–F). Further characterization of EPICs by sqPCR confirmed the expression of smooth muscle (α-SMA, γ-SMA) and fibroblastic markers (collagen I; prolyl-hydroxylase 4) in these cells (Fig. 3J). Although EPICs did not seem to differentiate into cardiac striated muscle or endothelial cells, they maintained the expression of some pre-cardiogenic (Gata4, Nkx2.5 and SRF, but not Mef2c, Fig. 3J) and endothelial-related transcription factors (SCL/Tal1, Fig.·3J). EPICs also expressed Wilms tumor suppressor gene transcription factor (Wt1) (Fig. 3J), characteristic of embryonic epicardium [29]. In accordance with the immunohistochemical data presented above (Fig. 2), proepicardial cells (E9.5) and embryonic epicardial (E11.5) cells expressed a diversity of markers for endothelial, smooth muscle, cardiac muscle and fibroblastic cells (Fig. 3J).

### Cell surface marker profiling

**Figure 4:**
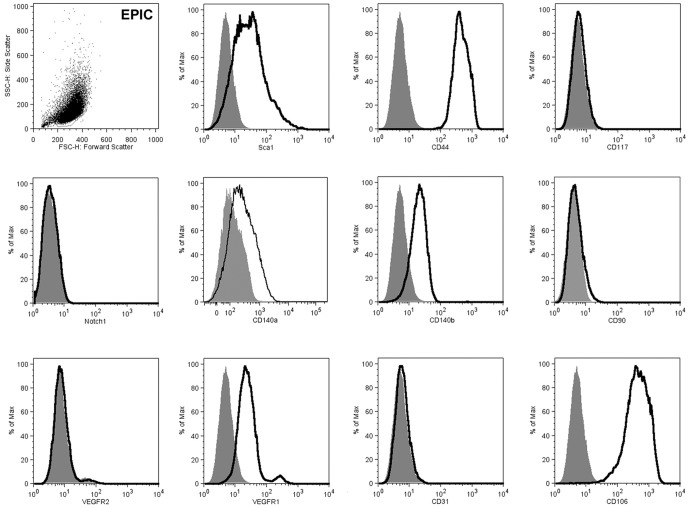
EPIC cell surface marker expression (FACS). EPIC expression of cell surface markers was evaluated by flow cytometry. Additional FACS analyses on ephrin and Eph receptors can be found in Fig. S4.

In order to characterize the EPIC line, we analyzed the expression of cell surface antigens by FACS (Fig. 4). While EPIC were positive for the stemness-like/progenitor markers Sca1, CD44, CD140a (PDGFRα; low expression), CD140b (PDGFRβ), they were negative for CD117 (c-Kit), and CD90 (Thy1). Although markers related to cardiovascular embryonic development like Flt-1 (VEGFR-1) and CD106 (VCAM) have been identified in EPIC, other markers which are continuously present on cells of the endothelial lineage (CD31/PECAM-1, Flk-1/VEGFR-2, Notch1) were absent. Finally, various ephrin ligands and Eph receptors have been found to be expressed by EPIC. In detail, EPICs were positive for ephrin receptors (Eph) EphB3, B4, A2, A4, but negative for EphA1, EphA3, EphA5, EphA6, EphA7, EphA8 and EphB2. Regarding the ligands, EphrinB1 and B2, but not ephrins A1, A2, A4, were present in EPIC. (Fig. S3).

### Proteolytic activity and sprouting capacity of EPIC

**Figure 5:**
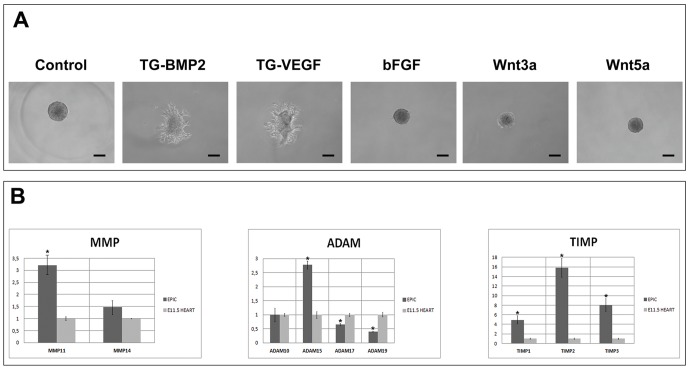
MMPs, ADAMs & TIMPs expression. **A.** EPIC spheroids cultured on regular fibrin gels (treated or un-treated with soluble bFGF, Wnt3a or Wnt5a) or on transglutaminase-bound BMP2 or VEGF fibrin gels for 48 hours. Matrix degradation is indicated by an halo around the cell spheroids. **B**. qPCR study of MMP, ADAM and TIMP expression levels. (p<0.05). Scale bars: 100 µm.

**Figure 6:**
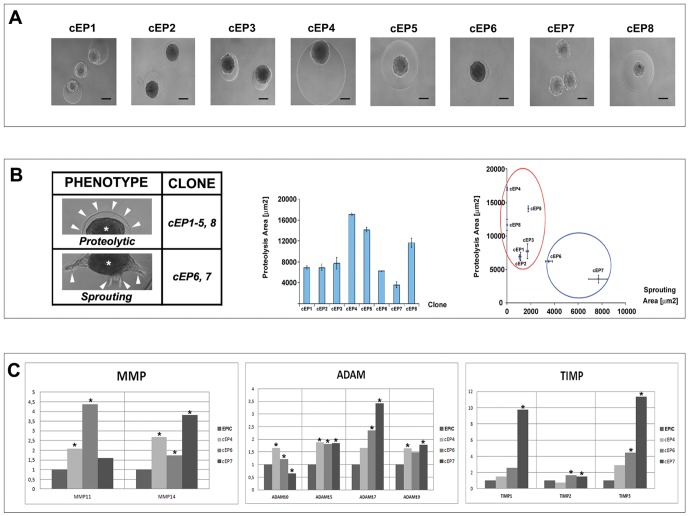
Evaluation of EPIC clones (cEP) proteolytic activity and sprouting. **A**. Representative images are shown for the culture of EPIC clones (cEP1–8) in 3D fibrin gels. **B.** The phenotype of the clones is illustrated in the left table. Note that some cell spheroids (asterisks) preferentially degrade the fibrin (‘proteolytic’ clones), generating characteristic halo around the cells (arrowheads). Others (‘sprouting’ clones) attach to the fibrin and spread over it forming multicellular sprouts (arrowheads). The fibrin gel digested area was graphically represented for each clone (middle) and plotted against the respective sprouting area of each clone (µm^2^, right). **C**. qPCR analyses of MMP, ADAM and TIMP expression in three significant cEP (cEP4 for maximal proteolysis and cEP6,7 for maximal sprouting). (p<0.05). Scale bars: 100 µm.

Embryonic EPDCs and cardiac fibroblasts are known to be able to migrate through and degrade the extracellular matrix (ECM). To test the proteolytic activity of the EPIC line we established a 3D culture assay in which EPIC spheroids generated by a classic hanging drop method (with EPICs suspended in methylcellulose containing medium) were cultured in growth factor-loaded fibrin gels microenvironments. Spherical aggregates of EPICs cultured in control fibrin gels actively degraded this matrix as illustrated by the formation of a proteolysis halo around the cells (Fig. 5A). When the spheroids were treated with soluble bFGF, Wnt3a and Wnt5a, EPICs showed a reduced proteolytic activity (as identified by the reduction of the proteolysis halo) (Fig. 5A). In an additional series of experiments, EPICs were grown within fibrin gels containing engineered growth factors (TG-BMP2 and TG-VEGF_121_), which are covalently tethered to the fibrin network by the human transglutaminase (TG) factor XIII [31], and gels without growth factors (Fig. 5A). TG-BMP2 and TG-VEGF_121_ decorated fibrin gels promoted the attachment, migration and spreading of EPICs without massive degradation of the gel (‘sprouting’ phenotype, see also Fig. 6B). Routine tests were performed to check whether EPICs differentiation into endothelium (VEGF treatment) [22] or cardiac muscle (BMP-2 treatment) [24] was occurring in fibrin gels with TG-bound growth factors. No differentiation into these cell types could be recorded (VE-cadherin, VEGFR2, myocardin, Mef2c sqPCRs, data not shown).

qPCR analysis of EPICs, as compared with E11.5 whole hearts, demonstrated a characteristic expression profile for a variety of molecules involved in the regulation of ECM proteolytic degradation, mostly MMPs, ADAMs, and TIMPs (Fig. 5B). The EPIC line preferentially expresses MMP-11, ADAM, 15 and TIMP-1, 2 and 3, displaying a decreased expression of ADAM 17 and 19 as compared to embryonic heart tissue. No differences were found for MMP-14 and ADAM-10 (Fig. 5B).

### Proteolytic activity and sprouting capacity of EPIC clones

Since different cellular cell phenotypes were identified in the EPIC line, various EPIC clones were isolated by critical dilution and 8 of them (cEP1–8) were selected for experimentation as based on their morphology and proliferative activity (Fig. 6A).

Cell spheroids from EPIC clones cultured in 3D fibrin matrices showed different behaviors (Fig. 6B, left). Some clones exhibited extraordinary proteolytic capacity, identified by the appearance of a matrix degradation halo around the cell spheroids. Proteolysis was visible as early as 2–3 h after embedding spheroids in 3D matrices, and the complete degradation of the embedding fibrin was effective within 2 to 5 days (measured by the contact of the cell spheroids to the plastic). Fibrin degradation was found to be fast in cEP4,5,8, slower in cEP1-3 and very slow in cEP6,7 (Fig. 6B, middle). Remarkably, cEP6,7 displayed a characteristic ‘sprouting’ response after 48 hours that was absent in clones cEP4,5 and cEP8 (data not shown). Cell proteolytic activity was evaluated by estimating the digested area around spheroids, and the different cell clones were plotted for proteolysis (Y axis) and sprouting (X axis) (Fig. 6B, right). We observed an inverse relation between sprouting and the ability to digest the fibrin matrix (Fig. 6B and Fig. S4). Cells with the highest proteolytic activity group together (clones cEP1–cEP5) in the same cluster, while cells with clear sprouting activity group in a different cluster (Clones cEP6 and cEP7) (Fig. 6B, right). In order to determine whether the differences observed in 3D matrix degradation of EPIC clones correlate with certain proteases, we performed MMP, ADAM and TIMP mRNA profile analyses by qPCR (Fig. 6C). For these analyses clones representing the most extreme proteolytic activity (cEP4), the highest sprouting activity (cEP7) and intermediate properties (cEP6) were selected. Three independent qPCR experiments were run, with GAPDH as a reference gene. The results from these analyses indicated significant changes in the expression of MMP-11 and 14; ADAM-10, 15, 17 and 19; TIMP-1, 2, and 3 between different clones (Fig. 6C). To confirm the proteolytic properties of EPICs, cEP4 cells were cultured on fibrin gels containing the protease inhibitor aprotinin. Proteolysis in these gels was significantly reduced in these cultures as compared to control (no aprotinin) ones. The supernatant from EPICS and cEP4 cultures was also used for zymography assays. The results independently confirmed the proteolytic properties of EPICs (Fig. S5).

In parallel, cEP1–8 cell spheroids were embedded in a two-layer engineered fibrin matrix with transglutaminase(TG)-bound-BMP-2 or -VEGF (Fig. S4A). Exposure to these engineered 3D fibrin gels did not result in any observable changes in the differentiation of these cells towards endothelial or cardiomyocyte lineages (data not shown). However, some clones, when exposed to matrix-bound TG-BMP-2 (cEP6, cEP7) or TG-VEGF_121_ (cEP7), displayed variable degrees of cell sprouting into the fibrin gel with fast outward cell migration (Fig. S4A). All the other clones did not show any sprouting response under these experimental conditions, but instead degraded the surrounding matrix after 48 hours in culture. Since most cell spheroids did not show a response to VEGF, we used as control HUVEC cells to demonstrate that TG-VEGF_121_ incorporated into fibrin gels is fully functional. Indeed, HUVEC cells seeded in matrix with TG-VEGF_121_ readily migrated from the original spheroids. To evaluate TG-BMP-2 signalling activity, both BMPRI expression and pSMAD phosphorylation were confirmed (not shown) (Fig. S4A). Finally, the most extreme ‘sprouting’ phenotype (cEP7) was chosen for a detailed profiling. cEP7 cells migrate in response to TG-BMP2 and TG-VEGF_121_, as well as to bFGF embedded within fibrin (Fig. S4B). Wnt3a and Wnt5a treatments reduced the proteolytic activity of EPIC as compared to control cultures. The sprouting area for cEP7 in response to different growth factors was plotted as fold differences with respect to untreated control sets (Fig. S4B).

## Discussion

In this study we have analyzed the phenotype and properties of the epicardium-derived component of cardiac interstitial cells (CICs). We have focused our research on this CIC subpopulation for three different reasons. First, because embryonic epicardial mesenchymal derivatives (EPDCs) pioneer the colonization of the cardiac interstitial space, remaining as part of the cardiac interstitium throughout adulthood [17], [18], [26], [32]–[34]. Since the cardiac interstitium becomes more complex with time, interstitial cells of epicardial origin are likely to be involved in the progressive recruitment of cells from different origins to the cardiac interstitium. Second, EPDCs are known to invade multiple cardiac tissues, differentiating into a variety of cell kinds [18], [19], [35], [36]. This phenomenon requires the active migration of EPDCs, and thus the activation of efficient mobilization and proteolytic programs. Third, some EPDCs have been shown to differentiate into CFs [18], a cell type responsible for the fibrotic ventricular remodeling that follows chronic cardiac infarction.

Due to the complex biology of CICs (including CFs), new *in vitro* models to study the diversity and behavior of these cells under normal and pathologic conditions are needed. Other works have reported the use of epicardial continuous cell lines derived from neonatal rat epicardium [37], [38] or mouse embryonic epicardium [39], [40]. However, in most cases, these cell lines retain a full epithelial phenotype and are a poor model for epicardial mesenchymal derivatives, which display unique migratory and proteolytic properties.

Our work uses a new immortalized embryonic epicardial cell line derived from ED11.5 mouse hearts (EPIC). Original embryonic epicardial epithelial cells explanted *in vitro* continuously proliferate and expand, acquiring a characteristic mesenchymal phenotype and expressing known mesenchymal markers like Sox9. We have however identified in our cell line a few, small clones of cells that display an epithelial-like phenotype (Pan-Cadherin+, ZO-1+) (Fig. 1). The appearance of such cells can be the result of the immortalization procedure, but also illustrate a dynamic phenotypical plasticity between embryonic epicardial epithelial cells and their mesenchymal derivatives.

Since embryonic (pro)epicardial cells have been reported to differentiate into various cell types [18], [19], [24], and thus suggested to be multipotent [29], we have evaluated the differentiation potential of the EPIC line. In order to do so, we have first compared EPICs with epicardial progenitor cells (proepicardium) and E11.5 embryonic epicardial cells to screen the differentiation potential of the cells along the proepicardial-epicardial-EPDC developmental continuum. Mouse epicardial progenitor cells (proepicardial cells) are shown to differentiate into endothelial and smooth muscle cells, cardiomyocytes and fibroblasts. In contrast, cultured E11.5 epicardial cells and EPICs only express markers for smooth muscle cells (α-SMA) and fibroblasts (FSP1), and seem to have lost their potential to spontaneously differentiate into endothelial cells (CD31) or cardiomyocytes (MF20) *in vitro*. These data could be interpreted as the result of a progressive restriction of the developmental multipotency of epicardial progenitor cells as they transform into epicardial cells and EPDCs. However, it is not clear whether the full differentiation potential of epicardial cells is truly lost or the experimental procedure we have used fails to promote the outgrowth and propagation of specific progenitor cell types from the explants, as we have shown is the case of CD31+ coronary epicardial progenitors. In this context it is important to emphasize that our mRNA expression studies show that some markers for endothelial cells (Scl/Tal1) and cardiac muscle progenitors (Nkx2.5; Gata4; Srf) are expressed by EPICs even if they do not terminally differentiate into these cell types. This suggests that the endothelial/cardiomyocyte differentiation potential of embryonic EPDCs is not fully abrogated in the EPIC line, a concept supported by its basal expression of Wt1, a marker for non-differentiated embryonic EPDCs [41]. It is thus tempting to speculate that epicardial mesenchymal derivatives could differentiate into endothelial cells or cardiomyocytes if instructed with the proper signals. The latter interpretation is in accordance with recently published results suggesting that thymosin β4-dependent reprogramming of adult epicardial cells (from a Wt1+ lineage) allows these cells to recapitulate their embryonic potential and to differentiate into endothelium, smooth muscle and cardiomyocytes [42], [43].

Our results indicate that EPICs robustly differentiate into myofibroblast-like cells (α-SMA), smooth muscle cells (α-SMA/γ-SMA/SM-22+) and fibroblasts (FSP-1, collagen I, prolyl-hydroxylase 4). Interestingly enough, the percentage of α-SMA/SM-22+ cells is low if compared with the extensive expression of α-SMA/SM-22+ in a high percentage of EPICs, and it is therefore possible that α-SMA+ cells both represent myofibroblasts and immature smooth muscle cells. In this respect, recent reports have indicated that the differential expression of PDGFRα and β [33], [34], [41] is pivotal to the segregation of fibroblastic and smooth muscle cell lineages, respectively, from a common pool of EPDC progenitor cells [34]. In this study we show that EPICs express both PDGFRα and β and could be a good model to study smooth muscle versus cardiac fibroblast differentiation. Moreover, we would like to propose that the ‘myofibroblastic’ phenotype of some activated CF, including the massive expression of α-SMA, could be related to the epicardial origin of such cells, which might share a common progenitor with some cardiac smooth muscle cells. In this scenario, the genetic and signaling embryonic programs regulating epicardial cell differentiation, like those dependent on differential signaling via PDGF receptors alpha and beta, could also be responsible for the modulation of CF phenotype in the adult life. Such phenotype is dynamic, as shown by the variable expression of fibroblasts markers like FSP-1 (expressed in a small proportion of EPICs) or collagen I (expressed by a higher number of EPICs).

In relation to the migratory properties of the EPIC line, we have described the expression of molecules involved in the active migration of EPDCs like ephrins and their Eph receptors [44] through FACS analysis (Fig. S3). Remarkably, EphA2+ human cardiac stem cells have been described to be dependent on this ephrin receptor to migrate in response to myocardial infarction [45], but it is not known if the stem cells described in this work derive from the epicardium, as suggested for other cardiac stem cells [35]. On the other hand, the segregated expression of ephrins and Eph receptors in epicardial derivatives and other cell types in the cardiac interstitium could be instrumental to build up a functional cardiac niche microenvironment for cardiovascular progenitor/stem cells.

EPICs also express a variety of cell surface markers related to EPDC/fibroblastic adhesion to the ECM [46] and cell mobilization/migration [47] like CD106/VCAM [34]. Additional analyses of standard markers for stem-like/progenitor cells indicate that EPICs are Sca1-positive but c-kit- and CD90-negative, an expression profile previously described for some CSC [48]. EPIC also express other markers associated with mesenchymal stem cells like CD44 [49]. These results are in agreement with a recent report supporting the epicardial origin of a population of cardiac stem cells [35].

As it can be inferred from the previous paragraph, migration of CICs necessarily involves active ECM proteolysis, but also an efficient attachment of cells to the matrix. Our experimental setting compares the response of EPICs to regular and engineered fibrin gels (with TG-bound growth factors). Covalent binding of growth factors like BMP2 and VEGF to fibrin enhances cell attachment and spreading (‘sprouting’) of EPICs to the matrix without its massive degradation, whereas culturing these same cells on regular fibrin gels rather activates a strong proteolytic response. Then, the addition of soluble, non fibrin-bound Wnt3a and 5a abrogates EPIC degradation of fibrin matrices in our experiments, suggesting a limited migratory ability for these cells. As a matter of fact, it has been reported that Wnts can inhibit the migration of rat cardiac fibroblasts [50]. All these data, taken together, indicate that a fine balance between different signals is necessary to promote an efficient and permissive degradation of the matrix so that cells can initiate migration.

Matrix metalloproteinases (MMPs), endogenous tissue inhibitors of metalloproteinases (TIMPs), and disintigrin and metalloproteinase (ADAM) family proteins are known to be the main agents of ECM remodeling [5]. Our EPIC line displays a high expression level of MMP-11, ADAM-15 and TIMP-1, 2 and 3 as compared with E11.5 whole heart tissue. EPICs also express MM-14 and ADAM-10, but at a similar level to that found in embryonic whole heart extracts. This proteolytic profile correlates with an effective degradation of fibrin gels *in vitro*. Deficiencies in TIMP-2 activity after myocardial infarction accelerates adverse myocardial remodeling due to enhanced MMP-14 activity [51]. These results may suggest that the response of different CICs to heart damage, specially that of CFs, is diverse, and may be linked to endogenous proteolytic programs that, in turn, could relate to the embryonic origin of CFs. In this regard, TIMP-2 and MMP-14 are known to be involved in the activation of pro-MMP-2 via the formation of a trimolecular complex at the cell surface [51], which might play multiple roles in ECM remodeling processes, including the promotion of 3D gels invasion by human mesenchymal stem cells [52] and various CD44+ cell lines [53]. It is thus reasonable to suggest that the whole proteolytic program active during pathologic ventricular remodeling could depend on the interaction of CF subpopulations of various origins with very different proteolytic potentials.

Since the heterogeneous morphology and molecular phenotype of EPICs suggests that different EPIC subpopulations could have different migratory properties, we decided to analyze the proteolytic profile of various EPIC clones. Functional fibrin matrix degradation assays indicate that some clones present a fast fibrin degradation rate (cEP4, 5, 8), whereas other clones degrade the matrix in a slow manner but have a patent sprouting activity (cEP6, 7). We have found that EPIC fibrin degradation and sprouting over the matrix are negatively correlated. We interpret that EPIC clones with a high proteolytic activity degrade the matrix so fast that they fail to progress in cell-to-matrix adhesion and subsequent migration (represented by the ‘sprouting’ phenotype). This hypothesis would support the concept of CF activation and involvement in ventricular remodeling as a result of the interaction of different CF subpopulations. The analysis of our results indicates that the EPIC clone showing the highest sprouting activity (cEP7) mainly expresses high levels of MMP-14, ADAM-17 and TIMP-1 and 3, whereas the clone with extreme proteolytic properties (cEP4) mostly activates ADAM-10, sustaining a most balanced expression of other molecules like ADAM-15 or 19 or TIMPs. Hence, the role of the whole cardiac interstitium as an interactive community of cells (some of them sustaining *de novo* myocardial differentiation or myocardial survival, others developing a stromal, feeder-like role) could be instrumental to define their functions in reparative responses of the damaged heart [54]. Still, more research is required to identify the subpopulations of CICs (of epicardial and non-epicardial origin) that drive massive fibrotic responses in the diseased heart.

In conclusion, this study indicates that EPICs retain the ability to differentiate into various cardiovascular cell kinds, especially those related to cardiac interstitium development (myofibroblasts and CFs). Furthermore, EPIC display a complex proteolytic program built from the interaction of the characteristic proteolytic properties of EPIC subpopulations. Finally, EPICs could be used as a good model to study ventricular remodeling by contributing to the identification of signaling pathways related to cardiac interstitium homeostasis and cell surface molecular profiles that could be used to characterize and isolate subpopulations of epicardial-derived CFs. This, in turn, could be instrumental to identify the roles that different CICs play in response to heart damage (i.e. fibrosis or active ECM degradation). A great variety of essential questions related to the maturation and response of CICs to episodes of hypoxia or inflammation remain open, and extensive and systematic research is required to develop new strategies to minimize cardiac fibrotic disease.

## Supporting Information

Figure S1 Coronary endothelial angioblasts/cells do not follow epicardial outgrowth in vitro. **A**. CD31 whole mount immunohistochemistry labels early subepicardial coronary angioblasts and endothelial cells (arrowheads). This kind of cell is absent from epicardial cell outgrowths in E11.5 whole heart explants (please, refer to **Fig. 2G,H**). **B-B′**. Microdissection of E11.5 mouse hearts in cold trypsin allows for the manual isolation of embryonic epicardial cells. Note that after this mechanical extraction, CD31+ angioblasts/endothelial cells can be found in epicardial explants in vitro (green). **C–D**. VEGFR-2 immunohistochemistry identifies vascular endothelium (asterisk, green) and angioblasts (arrowheads, green) in E13.5 mouse embryo samples (**C**), while EPICs remain VEGFR-2-negative (**D**). **E**. EPICs are immunoreactive to smooth muscle-specific myosin antibodies (red cells, arrowheads). Scale bars: A,B,C = 100 µm; B′,D,E = 50 µm.
(EPS)

Figure S2 Quantification of α and γSMA expression in TGFβ-induced EPIC cultures. Quantitative PCR confirms the increased expression of α- and γ-SMA in TGFβ1-treated EPICs (left). TGFβ2-treated cultures show an increased expression of γ-SMA but not α-SMA (p value<0.05).
(EPS)

Figure S3 Ephrin and Eph EPIC profiling. Expression of Ephrin ligand and ephrin receptor (Eph) in EPICs.
(EPS)

Figure S4 cEP behaviour on TG-fibrin matrices: proteolytic activity and sprouting. **A**. cEP spheroids show different proteolytic/sprouting responses when cultured in TG-BPM2 and TG-VEGF fibrin matrices as compared to control experiments (regular fibrin). HUVEC cells are shown as internal control for VEGF activity. **B**. cEP7 spheroids were embedded into a 3D fibrin matrix with TG-bound-BMP2 and -VEGF_121_ or soluble bFGF, Wnt3a, Wnt5a, and examined after 48 h. cEP sprouting quantification after the different treatments has been graphically presented. Scale bars: 100 µm.
(EPS)

Figure S5 cEP4 zymography and protease inhibitor assays. **A.** 10% SDS-PAGE gels with 1.5 mg/ml gelatin were used to run cell culture supernatants. Gelatin degradation (48 hours of zymographic reaction) is shown for media from cEP4, EPICs, and proper controls, including plain culture medium, plasmin and supernatant from HT1080 cells (HT1080 is a fibrosarcoma line known to express MMPs after TPA phorbol ester treatment). **B**. After 24 h cEP4 cells cultured on fibrin gels degrade the substrate and aggregate at the bottom of the culture dish (left, asterisk). Treatment with aprotinin reduces proteolysis and cells remain in the surface of the fibrin gel (arrowheads).
(EPS)
